# Development of a real-time QPCR assay for the detection of RV2 lineage-specific rhadinoviruses in macaques and baboons

**DOI:** 10.1186/1743-422X-2-2

**Published:** 2005-01-05

**Authors:** A Gregory Bruce, Angela M Bakke, Margaret E Thouless, Timothy M Rose

**Affiliations:** 1 Department of Pathobiology, School of Public Health and Community Medicine, University of Washington, Seattle, WA 98195 USA

## Abstract

**Background:**

Two distinct lineages of rhadinoviruses related to Kaposi's sarcoma-associated herpesvirus (KSHV/HHV8) have been identified in macaques and other Old World non-human primates. We have developed a real-time quantitative PCR (QPCR) assay using a TaqMan probe to differentially detect and quantitate members of the rhadinovirus-2 (RV2) lineage. PCR primers were derived from sequences within ORF 60 and the adjacent ORF 59/60 intergenic region which were highly conserved between the macaque RV2 rhadinoviruses, rhesus rhadinovirus (RRV) and *Macaca nemestrina *rhadinovirus-2 (MneRV2). These primers showed little similarity to the corresponding sequences of the macaque RV1 rhadinoviruses, retroperitoneal fibromatosis herpesvirus *Macaca nemestrina *(RFHVMn) and *Macaca mulatta *(RFHVMm). To determine viral loads per cell, an additional TaqMan QPCR assay was developed to detect the single copy cellular oncostatin M gene.

**Results:**

We show that the RV2 QPCR assay is linear from less than 2 to more than 300,000 copies using MneRV2 DNA, and is non-reactive with RFHVMn DNA up to 1 billion DNA templates per reaction. RV2 loads ranging from 6 to 2,300 viral genome equivalent copies per 10^6 ^cells were detected in PBMC from randomly sampled macaques from the Washington National Primate Research Center. Screening tissue from other primate species, including another macaque, *Macaca fascicularis*, and a baboon, *Papio cynocephalus*, revealed the presence of novel rhadinoviruses, MfaRV2 and PcyRV2, respectively. Sequence comparison and phylogenetic analysis confirmed their inclusion within the RV2 lineage of KSHV-like rhadinoviruses.

**Conclusions:**

We describe a QPCR assay which provides a quick and sensitive method for quantitating rhadinoviruses belonging to the RV2 lineage of KSHV-like rhadinoviruses found in a variety of macaque species commonly used for biomedical research. While this assay broadly detects different RV2 rhadinovirus species, it is unreactive with RV1 rhadinovirus species which commonly co-infect the same primate hosts. We also show that this QPCR assay can be used to identify novel RV2 rhadinoviruses in different primate species.

## Background

Members of the *Rhadinovirus *genus of the gammaherpesviruses are lymphotrophic and are associated with a variety of lymphoproliferative diseases. Herpesvirus saimiri (HVS), the prototype rhadinovirus isolated from the South American squirrel monkey, causes fulminant T-cell lymphomas in closely related host species [1]. Kaposi's sarcoma-associated herpesvirus (KSHV)/human herpesvirus 8, the only known human rhadinovirus, is associated with classical and AIDS-related Kaposi's sarcoma, primary effusion lymphoma and multicentric Castleman's disease [2]. Other rhadinoviruses have been isolated from ruminants, including wildebeest, sheep and cows, that are associated with malignant catarrhal fever, a lymphoproliferative syndrome [3,4].

We and others have demonstrated the existence of two distinct lineages of KSHV-like rhadinoviruses in Old World non-human primates [5,6]. The rhadinovirus-1 (RV1) lineage includes KSHV and closely related homologs infecting different Old World non-human primate species. In macaques, the RV1 lineage is represented by retroperitoneal fibromatosis herpesvirus (RFHV) that was identified in retroperitoneal fibromatosis (RF) tumor lesions of two macaque species at the Washington National Primate Research Center (WaNPRC) [7,8]. The RV2 lineage in macaques includes rhesus rhadinovirus (RRV) which was first identified in co-cultures of peripheral blood mononuclear cells (PBMC) of rhesus macaques (*M. mulatta*) in the New England National Primate Research Center (NENPRC) [9] and pig-tailed macaque rhadinovirus/*M. nemestrina *RV2 (MneRV2) [5,10,11]. While sequence analysis of the RRV genome demonstrated a close similarity to KSHV [12,13], phylogenetic analysis of multiple gene sequences has grouped RRV and the closely related MneRV2 within the RV2 lineage distinct from RFHV and KSHV [5]. Although RV2 rhadinoviruses have been identified in all Old World non-human primates tested, including gorillas and chimpanzees, no evidence of a human homolog has so far been found [6,14-17].

While complete genomic sequences have been obtained for two closely related strains of the RV2 lineage rhadinovirus of rhesus macaques, RRV strain H26-95 from the NENPRC [13] and RRV strain 17577 from the Oregon National Primate Research Center (ONPRC) [12], little information is known regarding the sequences of RV2 rhadinoviruses from other macaque species, and assays to detect these rhadinoviruses have not been developed. Quantitative real-time PCR assays (QPCR) have been developed to specifically detect RRV in rhesus macaque samples [18,19], but these assays have not be shown to cross to other RV2 rhadinovirus species. Since the WaNPRC and other primate research centers in the US and abroad utilize macaque species other than rhesus for biomedical research, we decided to obtain sequence information from the RV2 rhadinovirus of pig-tailed macaques, MneRV2, in order to develop a general assay to detect RV2 rhadinoviruses from different macaque species. Our strategy was to identify gene sequences that were highly conserved between different RV2 species but not conserved within macaque RV1 rhadinoviruses, such as RFHVMn or RFHVMm, which are sometimes found in conjunction with RV2 rhadinovirus infections. Previous nucleotide sequence information for MneRV2 consisted only of a region of the DNA polymerase which had significant sequence similarity with the macaque RV1 rhadinoviruses, and therefore was unsuitable for the desired assay [5]. We analyzed several regions of the RV1 and RV2 rhadinovirus genomes as targets for a general RV2 specific assay and identified the ORF 59/60 junctional region as a suitable target. This region was highly conserved within macaque RV2 rhadinovirus species but not within macaque RV1 rhadinoviruses. In this paper, we report the development of a sensitive and specific TaqMan QPCR assay and its use in detecting and quantitating RV2 rhadinoviruses from different primate species.

## Results

### Identification of the ORF 59/60 junctional region from the RV1 and RV2 rhadinovirus species from *Macaca nemestrina*, RFHVMn and MneRV2

**Figure 1:**
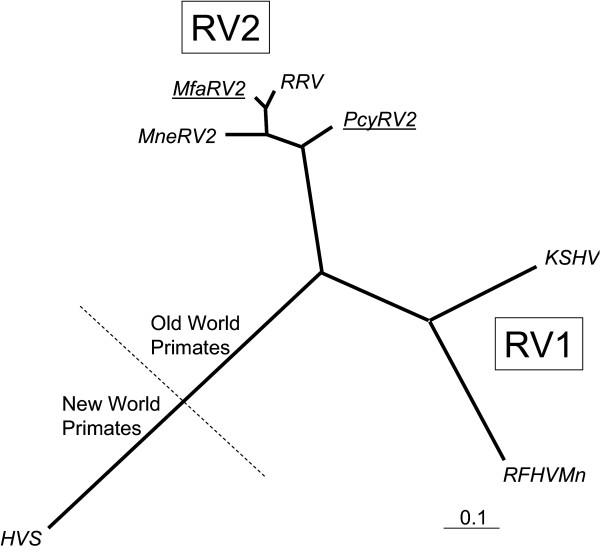
**Phylogenetic analysis of the nucleotide sequences of the ORF59/60 junctional region from various rhadinoviruses**. Sequences of the PCR products obtained using CODEHOP PCR primers from the rhadinoviruses MneRV2 (*M. nemestrina*), MfaRV2 (*M. fascicularis*), PcyRV2 (*Papio cynocephalus*) and RFHVMn (*M. nemestrina*) were aligned with the corresponding published sequences for KSHV (*homo sapiens*; U93872, bp 96678–97514), RRV (*M. mulatta*; AF083501, bp 92374–93205), and HVS (squirrel monkey, HSGEND, bp 81608–82613) using ClustalW. Phylogenetic analysis was performed using the DNA maximum likelihood procedure from Phylip. The division of New and Old World primate hosts is indicated. The RV1 and RV2 lineages of the Old World primate rhadinoviruses are shown. Novel viruses identified with the RV2 QPCR assay are underlined.

The ORF 59 and ORF 60 genes show high levels of homology between the related rhadinoviruses, KSHV and RRV, with 52% and 70% identity at the amino acid level, respectively [13]. Using the CODEHOP approach [20,21], we developed degenerate primers targeting conserved amino acid motifs "RDEL" (ORF 60) and "PQFV" (ORF 59) that would enable the amplification and sequence analysis of the ORF 59/60 junctional region of novel RV1 and RV2 rhadinovirus species as described in Materials and Methods. The CODEHOP primers were used in PCR amplification of DNA obtained from spleen tissue from 442N, a *M. nemestrina*, which has been previously shown to contain a co-infection of both MneRV-2 and RFHVMn rhadinoviruses [5]. PCR products from both MneRV2 and RFHVMn were obtained as described in Materials and Methods. Sequence analysis revealed a close similarity between the 833 bp of the ORF59/60 junctional region between the "RDEL" and "PQFV" motifs of MneRV2 and the corresponding region of RRV, with an 87% nucleotide identity. The 834 bp sequence of the RFHVMn junctional region was 67% identical to the corresponding region of KSHV and 60% identical to the RRV sequence. Phylogenetic analysis using DNA maximum-likelihood demonstrated a close clustering of the MneRV2 and RRV sequences, while the RFHVMn sequence clustered with the KSHV ORF59/60 sequence, as expected for the macaque homolog of KSHV (Figure 1).

### TaqMan quantitative PCR (QPCR) assay specific for RV2 rhadinoviruses

**Figure 2:**
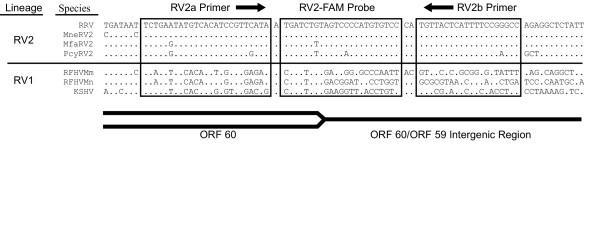
**Primer location and specificity of the RV2 QPCR assay**. Corresponding sequences from the end of ORF 60 and the adjacent intergenic region from different rhadinoviruses (see legend to Figure 1) were aligned. Rhadinovirus species and lineages are indicated. The primer set and probe were designed from the RRV and MneRV2 sequences. The RV2a primer and RV2-FAM probe were derived from the sense strand, as shown, while the RV2b primer was derived from the antisense strand. The alignment shows the mismatches between the primer and probe sequences and the MfaRV2 and PcyRV2 sequences identified with the RV2 assay in this study. Dots represent residues identical to those in the RRV sequence, and highlight the similarity of the primer sequences within the RV2 lineage of rhadinoviruses and the dissimilarity with members of the RV1 lineage of rhadinoviruses.

Multiple alignment of the RRV and MneRV2 nucleotide sequences revealed large regions of identical sequences within both the ORF 59 and ORF 60 coding regions and the ORF 59/60 intergenic region. As shown in Figure 2, a region of 71 identical nucleotides in the MneRV2 and RRV sequences was identified at the 3' end of the ORF 60 gene and the adjacent intergenic region. This region was only 43% conserved with the corresponding sequence of RFHVMn. Using Primer Express software (Applied Biosystems), a set of PCR primers (RV2a and RV2b) and a probe (RV2-FAM) were identified for a TaqMan QPCR assay (71 bp amplicon) which would specifically detect these macaque RV2 rhadinoviruses and not cross to known RV1 rhadinoviruses (Fig. 2 and Table 1).

**Table 1:** PCR primers

Primer^1^	Gene Target	Sequence^2^
**RV2 QPCR Assay (Figure 2)**		
RV2a	RV2 ORF 60	5'-TCTGAATATGTCACATCCGTTCATA-3'
RV2b	RV2 ORF 59/60 intergenic	5'-GGCCCGGAAAATGAGTAACA-3'
RV2-FAM^3^	RV2 ORF 60 and 59/60 intergenic	5'-(6-FAM)-TGATCTGTAGTCCCCATGTGTCC-(BHQ-1)-3'
**OSM QPCR Assay (Figure 3)**		
OSMa	Exon 3 OSM	5'-CCTCGGGCTCAGGAACAAC-3'
OSMb	Exon 3 OSM	5'-GGCCTTCGTGGGCTCAG-3'
OSM-FAM	Exon 3 OSM	5'-(6-FAM)-TACTGCATGGCCCAGCTGCTGGACAA-(BHQ-1)-3'
**ORF 59/60 CODEHOP Primers**		
RDELa^4^	ORF 60 bias^5 ^KSHV	5'-CTTGCCAACGATTACATTTCCAGRGAYGARCT-3'
SRDEa^4^	ORF 60 bias RRV	5' CTGGCTAACGACTACATCTCCAGRGAYGARCT-3'
NFFEa	ORF 60 bias KSHV	5'-GGCAGTTTCAAGGCTGTGAATTTYTTYGARCG-3'
PQFVb^6^	ORF 59 bias KSHV	5'-CCGTAAGAAATGGTGGTCCTGACRAAYTGNGG-3'
QFVRb^6^	ORF 59 bias RRV	5'-CCGTAGGCGATGGTCGTCCTAACRAAYTGNGG-3'
CFICb	ORF 59 bias RRV	5'-TACAAAATACAGCGAGTGATANATRAARCA-3'
**Gene-Specific Primer**		
MPVDb	ORF 59 (RFHV/KSHV)^7^	5'-TGAAAATCCACAGGCATGAT-3'

^1^The terminal "a" or "b" in the primer name indicates the plus or minus sense of the gene transcription, respectively.

^2^IUB code for ambiguous nucleotides: R = A or G; Y = C or T; N = A, C, G, or T

^3^FAM indicates a TaqMan dual-labeled probe with the fluorescent dye 6-FAM at the 5' end and the "black hole quencher" (BHQ) dye at the 3' end.

^4^These CODEHOP primers target the same motif but are biased differently (see below).

^5^"bias" indicates that the 5' consensus region of the CODEHOP primer was derived from a particular sequence" see [20].

^6^These CODEHOP primers target the same motif but are biased differently.

^7^This primer sequence is identical to the RFHVMn, RFHVMm and KSHV sequences

### TaqMan QPCR assay for the cellular gene, oncostatin M, to determine cell number

**Figure 3:**
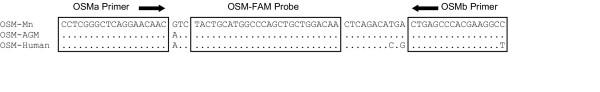
**Primer location and specificity for the OSM QPCR assay to detect cell copy number**. Corresponding sequences from the third exon of the OSM gene from human, African green monkey (AGM) and pig-tailed macaque (Mn) are aligned with the positions of the OSM primer set and probe indicated. The OSMa primer and OSM-FAM probe were derived from the sense strand, as shown, while the OSMb primer was derived from the antisense strand.

In order to determine viral copy number per cell, an additional TaqMan QPCR assay was developed to detect a single copy cellular gene, oncostatin M (OSM) [22]. We had previously determined the sequence of the OSM gene in an African green monkey which was highly conserved with the human gene (unpublished results). Using PCR primers derived from consensus sequences of the human and monkey gene, we determined the sequence of the entire OSM coding sequence of the *M. nemestrina *OSM gene (data not shown). Multiple alignment of the human, monkey and macaque OSM sequences revealed a region within exon 3 which was highly conserved. Using Primer Express software, a set of primers (OSMa and OSMb; 76 bp amplicon) and a probe (OSM-FAM) were identified (Fig. 3 and Table 1) which could be used to detect OSM DNA from macaque, monkey and human sources allowing quantitation of cell number in tissue samples.

### QPCR Assay Development and Characterization

The RV2 and OSM QPCR assays were optimized using DNA obtained from the spleen of a rhesus macaque, MmuA01111, which we have previously determined to contain RRV DNA in a background of macaque genomic DNA [23]. Initially, a temperature gradient PCR was performed to determine annealing temperatures that gave a single PCR product. An annealing temperature of 62°C was chosen because that temperature was optimal in both the RV2 and OSM assays (data not shown). The magnesium chloride, nucleotide, primer and probe concentrations were then varied to determine conditions which gave optimal efficiency.

**Figure 4:**
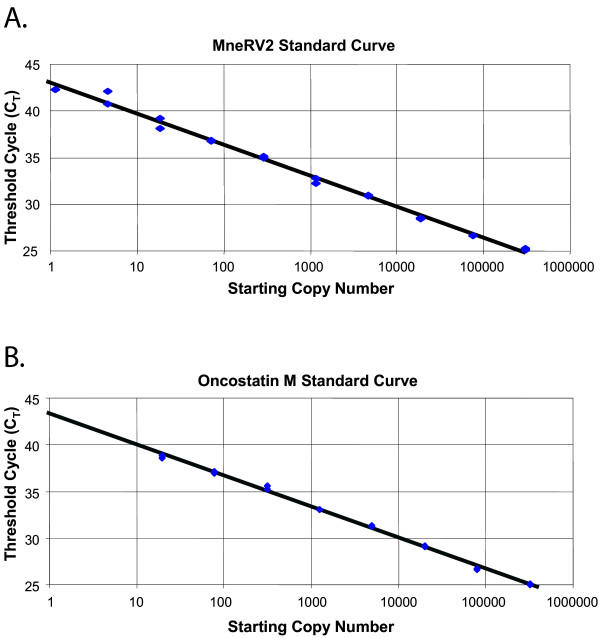
**Standard curves obtained from the RV2 rhadinovirus and OSM reference cellular gene assays**. A) The standard RV2 assay was performed on purified MneRV2 DNA in a series of four-fold dilutions over the range of 2 copies to 3.0 × 10^5 ^copies of MneRV2. (slope = -3.320, 100% efficiency; r^2 ^= 0.997). B) The standard OSM assay was performed on MmuA01111 spleen DNA in a series of four-fold dilutions over the range of 0.06 ng (20 diploid OSM gene copies) to 1 μg (3.2 × 10^5 ^diploid OSM gene copies). (slope = -3.322, 100% efficiency; r^2 ^= 0.999)

Standard curves were obtained from a dilution series using the optimal conditions for the RV2 and OSM assays as described in Material and Methods. For the RV2 assay, purified MneRV2 DNA obtained from a lytic infection of rhesus primary fetal fibroblasts (RPFF) was assayed in duplicate using 4-fold dilutions. As seen in Figure 4A, the assay was linear across a range of dilutions from less than 2 to more than 3.0 × 10^5 ^copies of MneRV2, with a slope of -3.320 (100% efficiency) and r^2 ^= 0.997. For the OSM assay, MmuA01111 genomic DNA was assayed in duplicate using 4-fold dilutions, with the amount of DNA tested ranging from 0.06 ng (corresponding to 20 diploid OSM gene copies: equivalent to 10 cells) up to 1 μg (corresponding to 3.2 × 10^5 ^diploid OSM gene copies: equivalent to 1.6 × 10^5 ^cells). The assay was linear across this range with a slope of -3.322 (100% efficiency) and r^2 ^= 0.999 (Fig. 4B).

**Figure 5:**
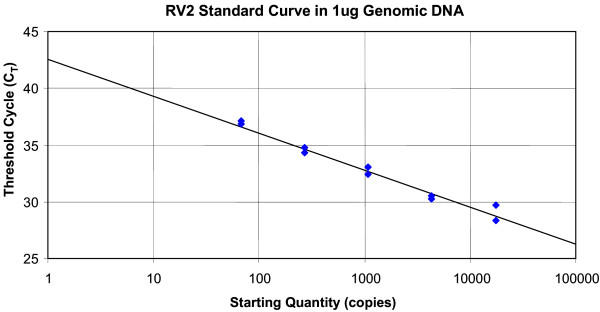
**Biologically relevant standard curve obtained with the RV2 rhadinovirus assay using RV2 DNA in a constant amount (1 μg) of genomic DNA**. DNA from MmuA01111 which was naturally infected with RRV was assayed in duplicate in four-fold dilutions made with uninfected macaque DNA. (slope = -3.318, 100% efficiency; r^2 ^= 0.988].

To determine the linearity of the RV2 assay with a biologically relevant sample, DNA from the spleen of MmuA01111 which contains cells naturally infected with RRV was subjected to 4-fold dilutions while keeping genomic DNA levels constant at 1 μg per reaction by the addition of DNA from an uninfected animal. The results demonstrate that the assay was linear from less than 66 copies of RRV (256-fold dilution of MmuA01111 DNA in uninfected macaque DNA) to more than 1.7 × 10^4 ^RRV copies per μg genomic DNA (MmuA01111 DNA undiluted) with a slope of -3.318 (100% efficiency) and r^2 ^= 0.988 (Fig. 5). This shows that the viral load determination would be accurate down to 410 RRV genomes/10^6 ^cells which is 1 viral copy per 2400 cells. The upper limit in this assay was determined to be greater than 110,000 viral genomes/10^6 ^cells which is the number of viral copies of RRV in 1 μg of DNA from the MmuA01111 spleen.

To ensure that the RV2 assay does not detect RV1 viruses, the assay was performed using DNA from the human and macaque RV1 rhadinoviruses. A DNA sample from the KSHV infected BCBL-1 cell line [24] containing approximately 4 × 10^6 ^copies of the KSHV genome and a sample containing 10^9 ^copies of a PCR product of the ORF59/60 junctional region from RFHVMn were used as templates in the RV2 assay. The RV-2 QPCR assay was negative for these templates under the standard reaction conditions.

### Identification of a novel RV2 rhadinovirus in *Macaca fascicularis *using the RV2 QPCR assay

Since the RV2 QPCR assay was based on consensus sequences shared by two distinct members of the RV2 lineage from *M. mulatta *and *M. nemestrina*, RRV and MneRV2, respectively, we tested to see if this assay could be used to identify a novel RV2 rhadinovirus in *M. fascicularis*. DNA was obtained from spleen tissue of Mfa95044, an *M. fascicularis *from the Tissue Distribution Program at the WaNPRC. Approximately 250 ng of spleen DNA produced a positive result in the RV2 QPCR assay with an average cycle threshold (C_T_) of 31.9 cycles. In order to prove that the assay detected a novel rhadinovirus, CODEHOP primers were used in a PCR amplification reaction with the Mfa95044 spleen DNA to obtain the ORF59/60 intergenic region of this rhadinovirus as described in the Materials and Methods. An 832 bp PCR product was obtained and sequenced. A comparison of this sequence with the corresponding region from RRV and MneRV2 showed 94% and 86% nucleotide identity, respectively. The nucleotide identity with the corresponding region in RFHV and KSHV was only 59% and 60%, respectively. Phylogenetic analysis showed a close clustering of the *M. fascicularis *sequence with the RRV sequence and a more distant relationship with the MneRV2 sequence, confirming its origin from an RV2 rhadinovirus of *M. fascicularis*, herein termed MfaRV2 (Figure 1). The evolutionary relationship of these rhadinovirus species mirrors that determined for the host macaque species themselves, where the *M. mulatta *and *M. fascicularis *have been shown to be more closely related to each other than to *M. nemestrina *[25]. Our data supports the hypothesis of a co-speciative divergence of the Old World primate rhadinoviruses and their hosts [26]

### Identification of a novel RV2 rhadinovirus in the baboon, *Papio cynocephalus*, using the RV2 QPCR assay

To further determine the specificity of the RV2 QPCR assay, DNA obtained from lymphocytes of baboon Pcy78404 was tested for the presence of a related RV2 rhadinovirus species under the standard assay conditions. Approximately 250 ng of lymphocyte DNA produced a positive result with an average C_T _of 33.8 cycles. In order to determine the identity of the reactive DNA species, CODEHOP primers were used in a PCR reaction with the baboon DNA as template as described in Materials and Methods. A product was obtained that yielded an 834 bp sequence which was 83% identical to the ORF59/60 intergenic region of each of the macaque RV2 rhadinoviruses, RRV, MneRV2 and MfaRV2, and 58% identical to the corresponding region in both KSHV and RFHVMn. The baboon sequence clustered with the macaque RV2 rhadinovirus sequences confirming its origin from an RV2 rhadinovirus of the baboon (*Papio cynocephalus*), herein termed PcyRV2. Phylogenetic analysis demonstrated that while PcyRV2 clustered within the RV2 rhadinovirus lineage, it branched off separately from the macaque RV2 rhadinoviruses as expected for a baboon rhadinovirus (Fig. 1).

Previously, an RV2 rhadinovirus, PapRV2, was detected in a baboon (*Papio anubis*) [27], and a partial sequence of the DNA polymerase was obtained. In order to compare PcyRV2 with PapRV2, we utilized CODEHOP PCR primers [7] to amplify a region of the polymerase gene of PcyRV2 that could be compared to the sequence available for PapRV2. DNA sequence for 352 bp of the DNA polymerase gene was obtained. An alignment of this sequence with the corresponding sequence of the PapRV2 rhadinovirus revealed a 97% sequence identity with 11 nucleotide differences which altered one amino acid.

### Specificity of the RV2 QPCR assay

**Figure 6:**
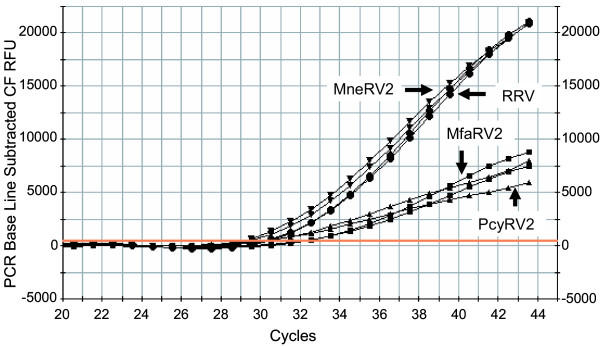
**Comparison of the RV2 QPCR assay using different rhadinovirus templates diluted in genomic DNA**. The PcyRV2 results were obtained using 1 μg of spleen DNA from baboon, Pcy78404, naturally infected with PcyRV2. The other rhadinovirus DNA templates were diluted in uninfected macaque genomic DNA to yield approximately equivalent C_T _values. The MneRV2 results were obtained using DNA from purified MneRV2 in macaque genomic DNA. The RRV results were obtained using DNA from spleen of MmuA01111, naturally infected with RRV. The MfaRV2 results were obtained using DNA from spleen of Mfa95044, naturally infected with MfaRV2. The released reporter fluorophore is plotted as a function of the amplification cycle number.

In order to compare the ability of the RV2 QPCR assay to detect different rhadinovirus templates, test samples containing roughly equivalent viral copy numbers in a background of genomic DNA were prepared. DNA from purified MneRV2, DNA from MmuA01111 spleen which contains RRV, and DNA from Mfa95044 spleen which contains MfaRV2 were diluted in DNA from a virus negative macaque to have approximately the same virus load as that found in the baboon lymphocyte DNA containing PcyRV2. As shown in Figure 6, all four samples have relatively similar levels of the different viruses, as indicated by the similar C_T _values (30.3, MneRV2; 30.8, RRV; 31.6, MfaRV2; and 33.2, PcyRV2). The cumulative fluorescence curve for the MneRV2 and RRV samples were superimposable with slopes typical of those seen in the assays performed in Figures 4 and 5 which showed amplification efficiencies of 100%. In contrast, both the *M. fascicularis *and baboon templates produced fluorescence curves with significantly decreased slopes, indicating lower amplification efficiencies. The efficiencies of these PCR reactions were calculated to be approximately 81% (r^2 ^= 0.900) for the MfaRV2 and 72% (r^2 ^= 0.929) for the PcyRV2, however, the low levels of virus in these samples made it difficult to accurately determine the efficiencies, as indicated by the correlation coefficients.

The novel ORF 59/60 intergenic regions of MfaRV2 and PcyRV2 were aligned with the corresponding sequences of RRV, MneRV2, RFHVMn, and KSHV. Also aligned was a partial sequence of the ORF 59/60 region obtained from RFHVMm (see Materials and Methods). As shown in Figure 2, the MfaRV2 sequence contained single nucleotide mismatches with the RV2a primer and RV2-FAM probe; an exact match was seen with the RV2b primer. The PcyRV2 sequence contained the same nucleotide mismatches seen in MfaRV2 and additionally had a second nucleotide mismatch within both the RV2a primer and the RV2-FAM probe. An additional mismatch was found between the PcyRV2 sequence and the RV2b primer. These nucleotide mismatches correlated with the decreased amplification efficiency of the assay with this template, as shown in Figure 6.

### RV2 QPCR screen of the prevalence of RV2 rhadinoviruses in macaques housed at the WaNPRC

DNA samples were obtained from PBMC of a random assortment of thirty macaques housed at the WaNPRC and analyzed using the standard RV2 and OSM QPCR assays. While all of the samples were positive for the single copy OSM gene, only six of the thirty macaques were positive for the presence of an RV2 rhadinovirus. In all of these six cases, both duplicate reactions in the assay were positive yielding average viral loads of 6–2300 per 10^6 ^cells (Table 2). However, in four of the six positive macaques, the RV-2 assay result was low and outside the linear range of the assay.

**Table 2:** RV2 rhadinovirus load in PBMC of 30 healthy macaques in the WaNPRC colony

Animal	RV2 DNA load in PBMC (Viral copies per 10^6 ^cells; mean ± SD^1^)
*M. nemestrina *(pig-tail)	
A98078	2300 ± 1200
F94132	650 ± 460
A98079	340 ± 49*
90152	5.8 ± 4.2*
16 other *M. nemestrina*	Below the limit of detection
	
*M. fascicularis *(crab-eating)	
98023	250 ± 96*
7 other *M. fascicularis*	Below the limit of detection
	
Unknown macaque species	
98062	57 ± 52*
1 other unknown species	Below the limit of detection
% of all macaques testing positive	6/30 = 20%

^1 ^Samples (1 μg) were assayed in duplicate and the means were determined. Standard deviations were calculated using the sum of the errors of the viral and OSM copy number determinations, as described in Materials and Methods.

* These results, while positive for both duplicates, were outside of the linear range of the assay.

## Discussion

We have developed a TaqMan probe-based QPCR assay to quantitate the viral load of macaque rhadinoviruses belonging to the RV2 lineage of KSHV-like rhadinoviruses. The primers and probe for this assay were based on sequences within the 3' end of the ORF 60 coding sequence and the ORF 59/60 intergenic region which were identical between the pig-tailed and rhesus macaque rhadinoviruses, MneRV2 and RRV, respectively, but were not conserved with the corresponding macaque viruses from the RV1 lineage of KSHV-like rhadinoviruses RFHVMn and RFHVMm. We have also developed a TaqMan probe-based QPCR assay targeting the single copy cellular gene, OSM, to serve as an internal control for quantitating cell copy number. Both assays were designed to give 100% PCR efficiency at the same annealing temperature, are linear over more than 4 orders of magnitude and are sensitive enough to detect less than 20 copies of the DNA target. The RV2 assay is able to accurately detect less than 66 copies of viral DNA in a genomic DNA background, even when the viral load is as low as 1 copy per 2400 cells.

Quantitation of the cellular DNA and viral DNA copy numbers in a tissue sample provides a suitable method for comparing viral loads, even between samples of unknown purity or degradation status. Because of the small size of the amplicons for both assays, OSM (76 bp) and RV2 (71 bp), viral loads can even be determined in formalin-fixed paraffin embedded tissue in which significant degradation of the DNA has occurred. Due to the similarities in sequence of the human, macaque and African green monkey OSM genes, the OSM QPCR assay may be suitable for quantitation of DNA in tissue from a number of other Old World primate species.

We have screened DNA from a number of random PBMC samples from macaques at the WaNPRC for the presence of an RV2 rhadinovirus. We detected RV2 rhadinovirus DNA in 6 of 30 macaques; 4 of 20 *M. nemestrina*, 1 of 7 *M. fascicularis *and 1 of 2 macaques whose species is not known. In these macaques, the viral copy number was determined to range from 6–2300 per 10^6 ^cells. Although the copy number in the single positive *M. fascicularis *was calculated to be 250 viruses per 10^6 ^cells, this would be a low estimate due to the 81% efficiency of the amplification of that template, as discussed above. Our results for RV2 rhadinoviruses in the macaque species tested at the WaNPRC were similar to those determined for RRV in rhesus macaques at the Tulane National Primate Research Center [18]. In the Tulane study, a QPCR assay developed against the interleukin-6 homolog of RRV found infrequent and low levels of RRV in PBMC of healthy and SIV-infected rhesus macaques. Only two healthy macaques had detectable RRV DNA with levels of 320 and 880 genomes per 10^6 ^cells. In the other 28 animals, the RRV load was below the level of detection. While RRV was detected more frequently in SIV-infected macaques in this study, the virus load was similar to that seen in healthy macaques.

The Tulane RRV assay had a similar sensitivity to our RV2 assay, with a lower limit of one RRV genome per 10,000 cell equivalents however, it was designed to specifically target only RRV while our RV2 assay is capable of detecting RRV, MneRV2 and other macaque and baboon rhadinoviruses. In this report, we have used the RV2 assay to detect novel RV2 rhadinovirus homologs in both the spleen of a crab-eating macaque (*Macaca fascicularis*) and the lymphocytes of a baboon (*Papio cynocephalus*). The standard RV2 assay had an amplification efficiency less than 100% with the *M. fascicularis *and *P. cynocephalus *templates which cautions against its use for accurate quantitation of the MfaRV2 and PcyRV2 rhadinoviruses. The primer and probe binding regions of these two rhadinoviruses showed nucleotide mismatches which correlate with the decrease amplification efficiency of the assay.

We have shown that the RV2 QPCR assay is capable of detecting a novel RV2 rhadinovirus, PcyRV2, in a baboon. Previously, an RV2 rhadinovirus, PapRV2, was also detected in baboons by others [27] using the degenerate PCR primer approach targeting the DNA polymerase gene that we had originally developed to detect novel herpesviruses [7]. In order to compare the two baboon viruses, we have sequenced a region of the DNA polymerase gene of PcyRV2. An alignment of this sequence with the corresponding sequence of the PapRV2 rhadinovirus revealed a 97% sequence identity with 11 nucleotide differences. This nucleotide similarity is consistent with the origin of these two viruses from two related species of baboons; the PcyRV2 rhadinovirus was isolated from the baboon species *Papio cynocephalus*, while the PapRV2 rhadinovirus was isolated from the baboon species *Papio anubis*.

## Conclusions

In this report, we describe a QPCR assay which provides a quick and sensitive method for screening RV2 rhadinoviruses found in the variety of non-human primate species commonly found in the National primate centers. While this assay broadly detects different RV2 rhadinoviruses species, it is unreactive with several RV1 rhadinovirus species. We also show that this QPCR assay can be used to identify novel RV-2 rhadinoviruses in primates.

## Methods and Materials

### Animals

Fresh frozen spleen tissue samples from *Macaca nemestrina *(Mne) 442N were provided by R. Shibata while at the National Institutes of Health, Bethesda, MD. This pig-tailed macaque had been experimentally infected with a pathogenic SHIV strain [28]. We have previously obtained PCR evidence for the presence of both RV1 and RV2 macaque rhadinoviruses, RFHVMn and MneRV2, respectively, in RF tumor and spleen tissue of this animal [5]. Fresh frozen RF tumor tissue from *Macaca mulatta *(Mmu) YN91-224, an SIV-infected rhesus macaque diagnosed with RF, was kindly provided by H. McClure, Yerkes National Primate Research Center. Fresh frozen spleen tissue samples were also obtained from *Macaca mulatta *(Mmu) A01111 at the WaNPRC, a rhesus macaque that had been experimentally infected with SIV which we have shown to be co-infected with the RV1 and RV2 macaque rhadinoviruses, RFHVMm and RRV, respectively (unpublished observations). Fresh frozen spleen tissue from a *Macaca fascicularis *(Mfa) 95044 and lymphocytes from a baboon (*Papio cynocephalus*) (Pcy78404) were kindly provided by H. Bielefeldt-Ohmann and C.-C. Tsai, respectively, from the WaNPRC. DNA from the PBMC of thirty random healthy colony macaques was also obtained from the virus screening program at the WaNPRC.

### Cells

The KSHV-infected pleural effusion lymphoma cell line, BCBL-1, was obtained from D. Ganem (Howard Hughes Institute – UCSF), and was carried in RPMI 1640 supplemented with 10% fetal bovine serum, penicillin, streptomycin, glutamine, and β-mercaptoethanol. Rhesus primary fetal fibroblasts (RPFF) were kindly provided by Dr. Michael Axthelm (ONPRC).

### Rhadinovirus

An isolate of MneRV2, was obtained from an *M. nemestrina*, MneJ97167, at the WaNPRC. The MneRV2 was used to infect cultures of RPFF and viral particles were harvested from culture supernatent by high speed centrifugation. Viral DNA used as positive controls in the PCR assays was obtained by disruption of the viral particles using phenol/chloroform and ethanol precipitation.

### DNA samples

DNA was extracted from frozen tissues using standard proteinase K-phenol/chloroform extractions and concentrated by ethanol precipitation.

### PCR amplification primers

The protein sequences of the ORF 59 and ORF 60 genes from KSHV and RRV were aligned using ClustalW. The consensus-degenerate hybrid oligonucleotide primer (CODEHOP) technique [20,21] was used to design two sets of degenerate PCR primers within both ORF 59 and ORF 60 that would enable the amplification and sequence analysis of the ORF 59/60 junctional region of novel RV1 and RV2 rhadinovirus species. The ORF 59 and ORF 60 genes are arranged in the same transcriptional orientiation in both RRV and KSHV. Two sense-strand CODEHOP primers, RDELa and SRDEa contained nucleotides encoding the highly conserved amino acid motif, Arg-Asp-Glu-Leu (RDEL; 8 fold degenerate), in ORF 60. Primer RDELa was biased toward the RV1 rhadinoviruses and contained a 5' consensus region derived from the KSHV sequence (Accession no. NC_003409). Primer SRDEa was biased toward the RV2 rhadinoviruses and contained a 5' consensus region derived from the RRV sequence (Accession no. AF210726). Two antisense-strand CODEHOP primers, PQFVb and QFVRb contained all coding possibilities for the highly conserved motif, Pro-Gln-Phe-Val (PQFV) in ORF 59 (16 fold degenerate), and were biased to the KSHV and RRV sequences, respectively (see Table 1). An additional anti-sense strand CODEHOP primer, CFICb (16 fold degenerate), was designed from a Cys-Phe-Ile-Cys (CFIC) motif in the ORF 59 gene, downstream of the PQFV motif and contained all coding possibilities for the CFIC motif and was biased to RRV.

### Amplification of the ORF 59/60 junctional region of novel rhadinoviruses

To obtain the ORF 59/60 junctional regions between the RDEL motif of ORF 60 and the PQFV motif of ORF 59 of MneRV2, PcyRV2, RFHVMn, and RFHVMm, DNA was obtained from different sources and used in PCR amplification with different CODEHOP PCR primers. Reactions were performed in 1 μM forward and reverse primers, 200 μM each dNTP, 20 mM Tris-HCl (pH 8.4), 50 mM KCl, and 2.5 units Platinum Taq polymerase (Invitrogen) using a 55–70°C annealing temperature gradient (BioRad Icycler). For MneRV2, PCR amplification was performed on Mne442N spleen DNA using primers RDELa and PQFVb. For PcyRV2, PCR amplification was performed on lymphocyte DNA from baboon Pcy78404, using SRDEa and QFVRb. In both cases an ~830 bp PCR fragment was obtained and sequenced. To obtain the sequence of RFHVMn which had a low copy number, it was necessary to amplify the RDEL-PQFV region in two fragments. A CODEHOP primer NFFEa (See Table 1), downstream of the RDEL motif was designed and used in conjunction with PQFVb to amplify an ~600 bp product from the Mne442N DNA. From the sequence of this product a specific primer, MPVDb, was derived and used in conjunction with RDELa to obtain an overlapping ~400 bp product. A similar strategy was used with RF tumor DNA obtained from MmuYN91-224 to obtain sequence from the ORF 59/60 junctional region of RFHVMm, however, only the sequence from NFFEA to PQFVB was obtained for comparison purposes. The ORF 59/60 junctional region of MfaRV2 was also obtained in two fragments. An ~400 bp PCR product was obtained after amplification of spleen DNA from Mfa95044, using the RV2 QPCR assay primer RV2b (see QPCR assay below and Table 1) and CODEHOP primer RDELa. An overlapping ~1400 bp PCR product was obtained using the RV2 QPCR assay primer, RV2a, in conjunction with an additional CODEHOP primer, CFICb.

### Sequence alignment and phylogenetic analysis

Nucleotide sequences were aligned using ClustalW and analyzed using the DNA maximum-likelihood program from the Phylip package, version 3.62 (University of Washington, Seattle). Phylogenetic tree output was produced using TreeView.

### Real-time QPCR design

The RV2 assay was designed to amplify a 71-bp amplicon from the ORF 59/60 junctional region of macaque viruses belonging to the RV2 rhadinovirus lineage using consensus primers "RV2a" (forward primer 5'-TCTGAATATGTCACATCCGTTCATA-3') and "RV2b" (reverse primer 5'-GGCCCGGAAAATGAGTAACA-3') with a TaqMan probe "RV2" 5'-(6-FAM)-TGATCTGTAGTCCCCATGTGTCC-(BHQ-1)-3' (Table 1 and Figure 1). As an internal control for cellular DNA which would allow the determination of the viral copy number per cell, a QPCR assay was developed to detect exon 3 of oncostatin M (OSM), a single copy cellular gene [Rose, 1993 #18]). The OSM assay amplifies a 76-bp amplicon from the macaque OSM gene using "OSMa" (forward primer 5'-CCTCGGGCTCAGGAACAAC-3') and "OSMb" (reverse primer 5'-GGCCTTCGTGGGCTCAG-3') with a TaqMan probe "OSM" 5'-(6-FAM)-TACTGCATGGCCCAGCTGCTGGACAA-(BHQ-1)-3' (Table 1 and Figure 2)

Reactions (50 μl) contained approximately 250–1000 ng of template DNA, 1 μM forward and reverse primers, 100 nM probe, 200 μM each dNTP, 20 mM Tris-HCl (pH 8.4), 50 mM KCl, and 2.5 units Platinum Taq polymerase (Invitrogen). Magnesium chloride concentrations were 4.0 mM for the RV2 assay and 2.0 mM for the OSM assay. After activation of the polymerase by incubation for 1 minute at 95°C, amplification was performed on a Bio-Rad iCycler equipped with an optical module for 45 cycles of 95°C for 30 s, 62°C for 30 s and 72°C for 30 s. The copy number for each assay was calculated from the cycle threshold (C_T_) determined using the Bio-Rad software. The viral load was calculated as a cellular genome copy equivalent by using the formula:

Viral load (genome equivalent copies) = Viral copy number/diploid OSM copy number

Samples were assayed in duplicate and the means were determined. Standard deviations were calculated using the sum of the errors of the viral and OSM copy number determinations.

## List of Abbreviations

AGM, African green monkey; CODEHOP, consensus-degenerate hybrid oligonucleotide primer; C_T_, cycle threshold; KSHV/HHV8, Kaposi's sarcoma-associated herpesvirus/human herpesvirus 8; Mfa, *Macaca fascicularis*; MfaRV2, *Macaca fascicularis *rhadinovirus-2; Mm/Mmu, *Macaca mulatta*; Mn/Mne, *Macaca nemestrina*; MneRV2, *Macaca nemestrina *rhadinovirus-2; ORF, open-reading frame; OSM, oncostatin M; Pcy, *Papio cynocephalus*; PcyRV2, *Papio cynocephalus *rhadinovirus-2; PCR, polymerase chain reaction; QPCR, quantitative PCR; RFHV, retroperitoneal fibromatosis herpesvirus; RRV, rhesus rhadinovirus; RV1, rhadinovirus-1; RV2, rhadinovirus-2;

## Competing Interests

The author(s) declare that they have no competing interests.

## Authors' Contribution

Design and conception of the study (AGB, TMR); development of the methods for amplification of the ORF59/60 regions (AGB, TMR); Development of the QPCR assays and quantitative analysis (AGB, AMB); Virus isolation and preparation (MET); Sequence analysis, alignment and phylogeny (AGB, AMB, TMR); Manuscript preparation (AGB, AMB, MET, TMR). All authors read and approved the final manuscript.
